# Children’s Respiratory Infections in Tianjin Area, China: Associations with Home Environments and Lifestyles

**DOI:** 10.3390/ijerph17114069

**Published:** 2020-06-07

**Authors:** Jing Hou, Dachao Lv, Yuexia Sun, Pan Wang, Qingnan Zhang, Jan Sundell

**Affiliations:** Tianjin Key Laboratory of Indoor Air Environmental Quality Control, School of Environmental Science and Engineering, Tianjin University, Tianjin 300350, China; jing_houj@163.com (J.H.); lvdachao914@tju.edu.cn (D.L.); panwang@tju.edu.cn (P.W.); qingnanstar@126.com (Q.Z.); sundellcc@gmail.com (J.S.)

**Keywords:** respiratory infections, perceived dry air, condensation, sun-cure bedding, modern floor covering

## Abstract

Children spend most of their indoors time at home, which may have substantial influence on their health. We conducted a cross-sectional study in the Tianjin area, China to quantify the incidence of respiratory infections among children, and its association with home environments and lifestyles. The lifetime-ever incidences of croup, pneumonia and ear infection among children aged 0–8 in Tianjin area was 9.2%, 28.7% and 11.6%, respectively. The incidence of common cold infections more than twice per year was 31.3%. Home environments and lifestyles included strong risk factors for childhood respiratory infections. Perceived dry air had the greatest association with childhood common colds (population attributable fraction (PAF = 15.0%). Modern floor covering had the greatest association with croup (PAF = 14.7%) and ear infection (PAF = 34.5%), while infrequent bedding sun-curing had the greatest association with pneumonia (PAF = 18.7%). Condensation (a proxy of poor ventilation) accounted for 12.2% of the incidence of croup (PAF = 12.2%) and frequent common colds (PAF = 8.4%). Our findings indicate that factors related to “modern” home environments and lifestyles are risks for childhood respiratory infections. Modifying such factors might reduce the incidence of respiratory infections among children.

## 1. Introduction

Respiratory infection was the leading cause of global children mortality, accounting for 15–16% of under-five mortality, second only to prematurity [1]. In 2017 alone, an estimated 1.6 million children under age 5 died worldwide from respiratory infections, including 39,000 deaths in China [2]. There is no evidence for a robust relationship between respiratory infections and genetic or racial factors [3]. People spend most of their time indoors, especially children [4,5]. Infants and young children, compared to adults, are more vulnerable to environmental exposure because their immune systems are not fully developed [6]. Household activities and environmental exposure at home are suspected risk factors for respiratory infections among children, especially in low income countries [7]. Therefore, a global study on home environments and children’s health was launched in eight countries/areas in 2000 [8,9], wherein children’s health outcomes, home environments, lifestyles, socialeconomic status, and biological factors were investigated systematically [10].

Home environmental exposures include chemical exposure (such as volatile organic compounds from building materials and decoration; NO_x_ from natural gas cooking), dampness and mold, and environmental tobacco smoke (ETS) pre- and post-natally. Many studies [11,12,13,14,15] have shown that dampness and mold in buildings are significantly associated with an increase in respiratory infections. People living in damp houses have been shown to be more likely to suffer from respiratory infections partly because of exposure to higher concentration of fungi and dust mites in damp environments [16,17]. Exposure to home indoor air pollutants such as ETS [18] and chemical sources [19] have also been reported to be associated with respiratory infections among children.

In addition to home environmental exposure, previous research has also shown that lifestyle factors and biological factors are important determinants of children’s health [20]. Sun-curing bedding and daily cleaning room are associated with fewer respiratory infections [21]. Holberg et al. [22] and Sun et al. [23] have demonstrated that daycare attendance was associated with more respiratory illness, asthma and allergy. Occupancy level and type of daycare facility have been identified as important determinants of infection risk [24,25]. The mode of infant delivery was found to affect early respiratory microbiota development, and may also play an important role in respiratory health later in life [26]. In addition, many studies in different countries have suggested that exclusive and prolonged breastfeeding was protective against respiratory infections [27,28,29].

With China’s increasing wealth and urbanization in the past 40 years, Chinese homes and lifestyles have changed dramatically. In urban areas, traditional Chinese residential buildings, Pingfang residences, have been replaced by high-rise apartments [10]. New furnishing materials have come into use [30]. Homes have been tightened in order to save energy, resulting in poor ventilation [31]. Along with this urbanization, a “modern” lifestyle has been adopted. It is less convenient for residents in urban areas to sun-cure bedding. Fast food has become popular. Childbirth tends to be cesarean delivery. Breastfeeding time has been reduced, and daycare is being started earlier. 

In view of these changes in home environments and lifestyles in modern China, the main aim of this article is to study associations of respiratory infection outcomes in children with their home environments and lifestyles.

## 2. Materials and Methods 

### 2.1. Questionnaire and Population

This study is part of the China, Children, Homes and Health (CCHH) project [9,32]. It was conducted from 2013 to 2015 in the Tianjin metropolis, that is, the city of Tianjin and its satellite city Cangzhou. The Tianjin metropolis is in northeast China, as shown in Supplementary Figure S1. Metropolitan Tianjin occupies an area of 11,920 km^2^ and has a population of 15 million. Cangzhou’s area is 13,420 km^2^ and its population is 7 million. In 2014, the GDPs per capita for Tianjin metropolis and Cangzhou were USD 16,500 and USD 5800, respectively [33]. The average outdoor air temperature for spring, summer, autumn and winter are 13.4 °C, 25.7 °C, 13.6 °C and −1.7 °C, respectively, in Tianjin and Cangzhou [34]. CCHH in Tianjin metropolis consisted of two phases: Phase I, a cross-sectional study and Phase II, a case-control study. In Phase I, we analyzed data obtained from a questionnaire survey of children’s health, demographic information, home environment and lifestyle. We selected daycares and primary schools from a list provided by the local Municipal Education Commission, through a stratified random sampling method. Finally, 24 institutes in urban, 6 in suburban and 9 in rural areas were involved in the survey. Questionnaires were sent to daycare centers and primary schools, from where they were delivered to parents who responded to the questionnaires. The details of the questionnaire are provided in Supplementary Materials—Questionnaire. The demographic information for investigated children included gender, age, family allergic history, home location and household income. Questions related to home environment were about indoor dampness; building characteristics and indoor furnishings; exposure to environmental tobacco smoke; and pets at home. Lifestyles referred to food habits; outdoor activity; home cleaning frequency; and children’s daycare attendance. We also investigated biological factors for children such as their delivery mode, birth weight; and length of breastfeeding.

Questions on respiratory infections are as follows:

- Has your child ever had croup? (possible responses: yes; no)
- Has your child ever had doctor diagnosed pneumonia? (possible responses: yes; no)
- Has your child ever had ear infections? (possible responses: no; yes, 1–2 times; yes, 3–5 times; yes, >5 times)
- In the last 12 months, how many times did your child have a common cold? (possible responses: none; 1–2 times; 3–5 times; 6–10 times; >10 times)

### 2.2. Statistical Analysis

All statistical analyses were performed with IBM SPSS Statistics 22 (International Business Machines Corporation (IBM), Armonk, NY, USA). We accepted *p*-values < 0.05 as statistically significant. We first analyzed children’s respiratory infections and their distribution by gender, age, family allergic history, home location and annual household income. The associations of childhood respiratory infections with home environments, lifestyles and biological factors were analyzed in univariate logistic regression models. Odds ratios were calculated in logistic regression models with adjustment for gender, age, family allergic history, home location, household income and outdoor pollution (indicated by PM_10_ concentrations, as shown in Supplementary Table S1). Then, correlation coefficients among factors that reached significant levels (*p* < 0.05) were investigated by Kendall correlation analyses. If the correlation coefficient was larger than 0.4, one factor in the pair was selected, and associations of the selected factors with respiratory infections were included in multivariate logistic regression models. Finally, we put all the significant predictors in multivariate logistic regression models (using a forward conditional method) to identify the most important risk factors for respiratory infections among children. 

Population attributable fraction (PAF) is an estimate of the fraction of population with the health outcome that can be attributed to a particular risk factor or exposure. We calculated PAF for a given exposure using the following formula [35]:

PAF = 1 − *P*_0_/*P_t_* (1)

where *P*_0_ is the cumulative proportion of unexposed persons who develop the disease over the interval and *P_t_* is the cumulative proportion of the total population developing disease over the specified interval. 

The Research Office at Tianjin University granted ethical approval for this study (No. 21207097).

## 3. Results

### 3.1. Demographic Information and Respiratory Infections in Children

We received 7865 questionnaires, a response rate of 78%. Ages were not reported for 204 children, while 295 children did not fit into the age range (0 to 8 years old). Therefore, there are 7366 children in the final analysis. Among these 7366 children, 52% were boys and 48% were girls. 

Table 1 shows that children’s respiratory infections had significant associations with family allergy history and home locations (*P* < 0.05). Croup was reported more often for boys. More wealthy urban children had more infections compared to less wealthy rural children. 

### 3.2. Home Environmental Factors Related to Respiratory Infections

#### 3.2.1. Dampness, Odors and Respiratory Infections

Dampness indicators and odors were defined as follows:

(1) Visible mold in child’s room;
(2) Visible damp in child’s room;
(3) Suspected moisture problem in child’s home;
(4) Peeling or discolored floor covering in child’s room;
(5) Flooding in child’s room;
(6) Condensation on windowpane in winter in child’s room;
(7) Perceived moldy odor;
(8) Perceived dry air.

Table 2 presents adjusted odd ratios (AOR) of dampness problems for respiratory infections. We found that living in a damp room was associated with increased odds of respiratory infections. Both pneumonia and the common cold were related to condensation on windowpanes. Perceived dry air was a significant risk factor for all respiratory infections among children.

#### 3.2.2. Building Characteristics and Respiratory Infections

The adjusted odds ratios of building characteristics for respiratory infections are presented in Supplementary Table S2. Infections (especially pneumonia) were associated with new apartments, modern floor covering, modern wall covering and use of air conditioners (AC). Meanwhile, exposure to new furniture and redecoration in the child’s early life due to home renovation was a risk factor for all respiratory infections. 

#### 3.2.3. Environmental Tobacco Smoke Exposure, Pet-Keeping and Respiratory Infections

The adjusted odds ratio of environmental tobacco smoke (ETS) and pet-keeping for respiratory infections are presented in Supplementary Table S3. Early life (i.e., during the first year of children’s life or during pregnancy) exposure to ETS, especially mother’s smoking, increased the risks of children’s respiratory infections. Pet-keeping was a risk factor for infections. 

### 3.3. Lifestyle Factors Related to Respiratory Infections

#### 3.3.1. Daycare Attendance and Respiratory Infections

The adjusted odds ratios of daycare attendance for the studied respiratory infections among children are presented in Supplementary Table S4. The type of childcare, starting age of daycare, exposure time and occupancy levels in daycare centers were analyzed. Children in daycare centers were more susceptible to pneumonia and common colds than children who were mostly at home. Children in daycare centers with more than 30 children have higher morbidity of pneumonia, ear infection and common colds.

#### 3.3.2. Food Habits, Outdoor Activity, Cleaning Habits and Respiratory Infections

Adjusted odds ratios for food habits, outdoor activity and cleaning habits for respiratory infections are presented in Table 3. Spending ≥3 h per day watching TV was a strong risk for the common cold. Children living in a frequently cleaned room with good ventilation and frequently sun-cured bedding had fewer respiratory infections.

### 3.4. Biological Factors

Table 4 presents adjusted odds ratios of biological factors for respiratory infections among children. Cesarean delivery is significantly associated with croup, pneumonia and ear infection. Children who were not born on due week with less birth weight had a higher morbidity of pneumonia.

### 3.5. Multivariate Regression Model and Population Attributable Fraction 

Factors that reached significant levels in univariate models were tested for correlation, as shown in Supplementary Table S5. Mold spots were correlated to damp spots. Modern floor covering was correlated to building type. Current pet keeping and environmental tobacco smoke were correlated to early life status. One factor in each of these paired variables was selected to be added to multivariate logistic regression models.

Finally, the variables mold spot, suspected moisture, floor moisture, condensation, moldy odor, perceived dry air, floor covering, cooling system, home renovation, early life smoking exposure, early life pets keeping, childcare type, size of daycare center, TV watching, room cleaning frequency, sun-curing bedsheets frequency, way of delivery, birth week and birth weight were used in the multivariate logistic regression model. Multivariate analysis of associations of respiratory infections with these home environment, lifestyle and biological factors is shown in Table 5. Dampness, condensation on windowpanes, moldy/perceived dry air, modern decoration materials and less frequency of sun-curing bed sheets were the greatest risk factors for croup, pneumonia, ear infections and common colds. In addition, attending day-care in a large class increased the risk of common colds and ear infections.

The population attributable fractions (PAFs) of home environment, lifestyle and biological factors for infections among children are shown in Table 6. The top contributor to childhood common colds is perceived dry air, while it is infrequent sun-curing bedding for pneumonia, and modern wall covering for croup and ear infection. Condensation, a secondary attribution factor, contributes 12.2% and 9.2% to croup and frequent common colds, respectively. Large class size is a risk factor for ear infection and common colds. 

## 4. Discussion

In this study, a multivariate regression model was applied to identify the greatest home environmental, lifestyle and biological risks for children’s infections. It was found that modern floor covering, perceived dry air (a proxy of indoor pollution), condensation on windowpanes (a proxy of poor ventilation), less sun-curing bedsheets and cesarean delivery are significantly associated with childhood infections. These factors might affect the development of the immune system and/or the transmission of infectious pathogens.

With respect to biological factors, we found that cesarean delivery and not being born on the due day were significant risk factors with high population attributable fractions (PAF) for childhood pneumonia. Bosch et al. pointed out that cesarean delivery affects early respiratory microbiota development, thereby possibly increasing the frequency of respiratory infections later in life [26]. In our study, the cesarean delivery rate was 68% in Tianjin metropolis, higher than the national level of 46% [36] and the recommended value by WHO of 15% [37]. Our previous study showed that the Tianjin modern urban area had a significantly higher cesarean delivery rate compared to the rural area close to Tianjin [10].

In addition to cities having a higher cesarean delivery rate, city dwellings have increasingly modern materials being used for indoor furnishings and decoration [38]. China is currently the largest producer of wood-based panels, coating and furniture in the world. Numerous new building furnishing materials have emerged in people’s daily life. In the present study, we found that children who live in an urban apartment decorated with modern floor and wall coverings were more likely to have had respiratory infections. Renovation during the first year of children’s life was also a risk. Considerable evidence has demonstrated that the chemical emissions of modern indoor materials (new wall coverings, new furniture, new synthetic carpets) are associated with increases in respiratory infections among children or infants [19]. Home renovation and modern decoration materials are positively associated with high concentrations of formaldehyde, volatile organic compounds (VOCs) and semi-VOCs [39,40]. These indoor pollutants may be associated with developmental delays in children, reduced activity of the immune system and direct toxicity [41,42]. As a comparison, suburban or rural children who lived in Pingfang dwellings with less “modern” redecoration and materials had fewer respiratory infections.

It is interesting to find that perceived dry air had strong associations with pneumonia and common colds. The reported rate of “perceived dry air” is high in Tianjin-53.1% in our study. “Perceived dry air” has been shown to not necessarily be due to physically dry indoor air, but rather to polluted air [43,44]. In spaces with poor ventilation, pollutants could not be efficiently removed [45], which might irritate the respiratory tract and reflect in more complaints of dry air [44].

In this study, condensation on the child’s room windowpanes in winter was a strong risk for respiratory infections among children. Condensation on windowpanes in winter has been reported to be associated with insufficient ventilation in homes [31,46]. Chinese homes do not have a mechanical ventilation system. Most of residential buildings in China still depend on the “natural ventilation” of open windows and infiltration to passively introduce outdoor air to indoors. In Tianjin, ~90% of homes closed the child’s bedroom window at night in winter. Thus, ventilation is mainly through infiltration, with a median value of 0.30 h^-1^ [31]. In such tight buildings, the poor ventilation rate could not efficiently dilute airborne-transmitted pathogens and subsequently results in their accumulation in our occupied spaces. A study conducted in army trainees found that rates of febrile acute respiratory diseases were significantly higher among trainees in modern (energy efficient design) barracks (an adjusted relative risk of 1.51) [47]. Milton estimated that in offices with lower ventilation, relative risk for short-term sick leave was 1.53 (95% CI 1.22–1.92) [48]. A study conducted in Chinese students’ dormitories indicated a clear dose–response relationship between ventilation rate and common cold infections [49].

Consistent with studies in America [50] and Sweden [51], our analysis demonstrated that daycare attendance was a significant risk factor for common colds among children in Tianjin. Daycare occupancy level and weekly exposure time are measures of contact with other children. We found that children spending more time in daycare or in higher occupancy daycare centers were susceptible to pneumonia, ear infections and common colds. Attending daycares with higher occupancy level was a contributor for common cold infections (Table 6, PAF = 9.0%), next to poor ventilation in homes (indicated by condensation on windowpanes [52]). More frequent contact with people may be a reasonable explanation for this observation. For respiratory infection viruses that are transmitted by small particle aerosols such as influenza, adenovirus, rhinovirus, and coronavirus, airborne transmission is considered the predominant transmission pathway [53,54]; however, contact (direct or indirect) can also spread these pathogens [55]. A study on the transmission of common colds in offices indicated that workers sharing offices have a significant risk of common colds compared to those working in single rooms (adjusted odds ratio—1.35) [56]. Overcrowding in a large urban jail was reported to be a significant risk factor (*p* = 0.03) for an outbreak of pneumococcal disease among inmates [57].

Currently in China, approximately 250 million families (accounting for 55% of the population in China) live in cities. Rapid urbanization and modernization has led China to experience a dramatic change in both indoor environments and lifestyles in the past two decades [30]. Modern high-rise apartments are constructed tightly and decorated with modern materials. The result is insufficient ventilation which causes increased concentrations of indoor-generated pollutants, including chemical compounds and airborne transmitted pathogens. It is almost impossible to sun-cure bedding frequently in modern urban buildings. It is common for children to attend daycare centers before three years old as both parents work in most cases. Meanwhile, more health issues related to indoor environments are being observed. Respiratory infections (as in this article), asthma and allergies [10] are more frequent in modern society, especially in homes with higher annual incomes (see Table 1). In a previous study, we showed that indoor pollution increased significantly with increasing household income [40]. Wealthier homes had more modern decoration materials (such as laminated wooden flooring) and household chemical products, all of which were associated with higher concentrations of modern chemical compounds (e.g., phthalates) [40]. As societies develop economically, ways to live a high-quality and healthy life need to be studied.

Parallel CCHH studies performed in other cities have found similarly high rates of respiratory infections in children, especially in urban areas [21,32]. The incidence of lifetime-ever pneumonia among children in modern cities (like Tianjin—28.7%) are substantially higher than those in USA and European cities [58]. Pneumonia is an infectious disease caused by bacteria or virus. The high infection rate of pneumonia in China may be partially due to insufficient vaccine coverage with the pneumococcal conjugate vaccine (PCV) and the Hemophilus influenzae type b (Hib) vaccine [58]. However, the incidence and severity or duration of pneumonia could be influenced by environmental factors [59]. Our analysis in the present study is hierarchical so as to identify the important factors among a variety of possible risk factors. The most prominent risk factors from multivariate analysis were related to modern home environments and lifestyles. The higher rates of childhood respiratory infections are linked with a combination of biological, environmental and behavioral factors, which affect the development of immune system and the breeding and transmission of pathogens.

In this study, surveys were performed using a stratified random sampling method. The response rate was 78%, which reduces the likelihood of selection bias. Therefore, the strong association of infections with home environments and lifestyles is unlikely to be due to bias.

## 5. Conclusions

Modern floor covering, perceived dry air, condensation on the windowpanes in winter, and infrequent sun-curing of bedding are the strongest risks for childhood respiratory infections. “Modern” home environments and lifestyles play an important role in the incidence of respiratory infections among children. Increasing ventilation rate, frequent sun-curing of bedding and avoiding pollutants from furnishing and decoration materials might be effective measures to reduce the incidence of childhood respiratory infections.

## Figures and Tables

**Table 1:** Respiratory infections among children in Tianjin, China, n (%).

		Croup		Pneumonia		Ear Infection		Common Cold >2 Times	
		Lifetime-Ever Incidence	*p*-Value ^a^	Lifetime-Ever Incidence	*p*-Value	Lifetime-Ever Incidence	*p*-Value	Incidence Per Year	*p*-Value
**Total**		**625 (9.2)**	**/**	**1959 (28.7)**	**/**	**794 (11.6)**	**/**	**2044 (31.3)**	**/**
**Gender**									
	Male	364 (10.4)	**0.000**	1043 (29.8)	0.064	411 (11.7)	0.707	1059 (31.8)	0.445
	Female	254 (7.9)		898 (27.8)		373 (11.4)		964 (30.9)	
**Age**									
	0–2	21 (10.0)	0.755	52 (24.0)	0.227	9 (4.3)	**0.001**	70 (36.5)	**0.000**
	3–5	268 (8.9)		878 (29.3)		378 (12.5)		1112 (38.8)	
	6–8	336 (9.4)		1029 (28.6)		407 (11.2)		862 (24.9)	
**Family allergic history**									
	Yes	156 (26.9)	**0.000**	421 (45.3)	**0.000**	176 (19.1)	**0.000**	392 (43.8)	**0.000**
	No	433 (7.8)		1436 (25.8)		584 (10.4)		1575 (29.7)	
**Home location**									
	Urban	375 (11.0)	**0.000**	1202 (35.2)	**0.000**	545 (16.0)	**0.000**	1137 (33.8)	**0.000**
	Suburban	107 (7.7)		333 (23.8)		130 (9.2)		406 (30.2)	
	Rural	105 (6.5)		331 (20.5)		75 (4.5)		387 (26.3)	
**Annual household income, $**									
	<4225	89 (8.7)		239 (23.1)		73 (7.0)		219 (23.0)	
	4225–7041	101 (7.2)		380 (27.0)		121 (8.5)		383 (28.6)	
	7042–14,082	163 (9.1)		597 (33.3)		242 (13.4)		612 (35.2)	
	14,083–28,164	159 (11.8)		516 (38.3)		189 (14.1)		457 (34.8)	
	>28,164	69 (12.5)	**0.000**	202 (35.9)	**0.000**	94 (16.8)	**0.000**	199 (36.4)	**0.000**

^a^*p*-value in Pearson chi-square test. Bold indicates statistical significance.

**Table 2:** Associations of respiratory infections with dampness and odors.

Adjusted Odds Ratio (AOR), 95% Confidence Interval (CI)				
	Croup	Pneumonia	Ear Infection	Common Cold >2 Times
**Mold spot in child’s room**				
No	1.00	1.00	1.00	1.00
Yes	**2.42 (1.64, 3.56)**	1.23 (0.90, 1.69)	**1.52 (1.01, 2.30)**	**1.42 (1.03, 1.94)**
**Damp spot in child’s room**				
No	1.00	1.00	1.00	1.00
Yes	**2.06 (1.46, 2.89)**	**1.34 (1.03, 1.73)**	**1.64 (1.16, 2.32)**	1.25 (0.96, 1.62)
**Suspected moisture in room**				
No	1.00	1.00	1.00	1.00
Yes	**1.56 (1.23, 1.97)**	**1.48 (1.26, 1.73)**	**1.46 (1.18, 1.81)**	**1.30 (1.10, 1.52)**
**Floor moisture in child’s room**				
No	1.00	1.00	1.00	1.00
Yes	**1.61 (1.10, 2.37)**	**1.37 (1.04, 1.80)**	1.34 (0.92, 1.95)	**1.40 (1.06, 1.85)**
**Flooding in child’s room**				
No	1.00	1.00	1.00	1.00
Yes	1.76 (0.95, 3.28)	1.53 (0.95, 2.48)	1.40 (0.75, 2.59)	1.45 (0.88, 2.37)
**Condensation on the windowpane in child’s room**				
No	1.00	1.00	1.00	1.00
Yes	**1.53 (1.23, 1.91)**	**1.34 (1.16, 1.55)**	1.09 (0.88, 1.34)	**1.60 (1.38, 1.84)**
**Perceived moldy odor in home**				
No	1.00	1.00	1.00	1.00
Yes	**1.72 (1.35, 2.20)**	**1.34 (1.13, 1.59)**	**1.81 (1.44, 2.28)**	**1.24 (1.04, 1.48)**
**Perceived dry air in home**				
No	1.00	1.00	1.00	1.00
Yes	**1.41 (1.16, 1.71)**	**1.28 (1.14, 1.45)**	**1.32 (1.11, 1.57)**	**1.43 (1.27, 1.62)**

Adjusted for gender, age, family allergic history, annual household income, outdoor PM_10_ (particles smaller than 10 microns) and home location. Bold indicates statistical significance.

**Table 3:** Associations of respiratory infections among children with dietary habits, outdoor activity, cleaning habits, sun-curing bed sheets and window-opening behavior.

Adjusted Odds Ratio (AOR), 95% Confidence Interval (CI)				
	Croup	Pneumonia	Ear Infection	Common Cold > 2 Times
**Fruit consumption**				
Occasionally/never	1.00	1.00	1.00	1.00
≥once/week	0.60 (0.32, 1.13)	0.70 (0.45, 1.08)	0.65 (0.34, 1.21)	1.35 (0.80, 2.25)
**Fast food consumption**				
Occasionally/never	1.00	1.00	1.00	1.00
≥once/week	1.09 (0.85, 1.40)	1.08 (0.92, 1.27)	1.05 (0.84, 1.32)	0.96 (0.81, 1.13)
**Watching TV**				
<3 h per day	1.00	1.00	1.00	1.00
≥3 h per day	1.37 (0.97, 1.94)	1.09 (0.86, 1.38)	1.18 (0.83, 1.68)	**1.39 (1.10, 1.75)**
**Outdoor activity**				
≤2 times per week	1.00	1.00	1.00	1.00
>2 times per week	1.18 (0.97, 1.43)	0.94 (0.83, 1.06)	1.00 (0.84, 1.19)	1.03 (0.91, 1.16)
**Cleaning room**				
Everyday	1.00	1.00	1.00	1.00
Less than everyday	1.09 (0.89, 1.33)	1.12 (0.98, 1.27)	**1.46 (1.22, 1.73)**	1.13 (0.99, 1.29)
**Opening window**				
Everyday	1.00	1.00	1.00	1.00
Less than everyday	1.00 (0.67, 1.50)	1.04 (0.81, 1.33)	0.76 (0.50, 1.15)	1.01 (0.78, 1.30)
**Sun-curing bed sheets**				
Often	1.00	1.00	1.00	1.00
Not often	**1.28 (1.04, 1.57)**	**1.46 (1.28, 1.65)**	**1.53 (1.27, 1.85)**	**1.28 (1.13, 1.45)**

Adjusted for gender, age, family allergy history, annual household income, outdoor PM_10_ (particles smaller than 10 microns) and home location. Bold indicates statistical significance.

**Table 4:** Associations between children’s biological factors and respiratory infections.

Adjusted Odds Ratio (AOR), 95% Confidence Interval (CI)				
	Croup	Pneumonia	Ear Infection	Common Cold >2 Times
**Way of delivery**				
Natural	1.00	1.00	1.00	1.00
cesarean	**1.22 (1.00, 1.48)**	**1.28 (1.13, 1.44)**	**1.25 (1.04, 1.49)**	0.96 (0.85, 1.09)
**Period of breastfeeding**				
>6 months	1.00	1.00	1.00	1.00
<6 months	1.08 (0.88, 1.33)	1.12 (0.98, 1.28)	1.07 (0.89, 1.29)	0.87 (0.76, 1.00)
**Born on due week**				
Yes	1.00	1.00	1.00	1.00
No	1.16 (0.96, 1.41)	**1.28 (1.13, 1.45)**	1.07 (0.90, 1.27)	0.98 (0.87, 1.12)
**Birth weight**				
>2.5 kg	1.00	1.00	1.00	1.00
<2.5 kg	0.91 (0.58, 1.42)	**1.33 (1.03, 1.71)**	1.30 (0.91, 1.86)	1.11 (0.85, 1.45)

Adjusted for gender, age, family allergy history, annual household income, outdoor PM_10_ (particles smaller than 10 microns) and home location. Bold indicates statistical significance.

**Table 5:** Multivariate analyses on associations of respiratory infections among children with home environmental, lifestyle, and biological factors in Tianjin, China.

Adjusted Odds Ratio (AOR), 95% Confidence Interval (CI)				
	Croup	Pneumonia	Ear Infection	Common Cold >2 Times ^m^
Floor moisture ^a^			2.02 (1.18, 3.43)	2.17 (1.29, 3.64)
Condensation ^b^	1.45 (1.07, 1.96)			1.55 (1.24, 1.94)
Perceived moldy odor ^c^	1.53 (1.03, 2.28)			
Perceived dry air ^d^		1.23 (1.02, 1.47)		1.49 (1.21, 1.84)
Modern floor covering ^e^	1.51 (1.08, 2, 12)	1.39 (1.15, 1.68)	1.54 (1.14, 2.09)	
Renovation, early ^f^	1.67 (1.07, 2.62)			
Environmental tobacco smoke (ETS) exposure, early ^g^		1.28 (1.07, 1.53)		
Daycare attendance ^h^				1, 47 (1.02, 2.12)
Size of daycare center ^i^			1.47 (1.15, 1.88)	1.41 (1.15, 1.73)
Sun-curing bedding ^j^		1.28 (1.07, 1.55)	1.69 (1.28, 2.23)	
Cesarean delivery ^k^		1.35 (1.11, 1.64)		
Born on due week ^l^		1.33 (1.10, 1.59)		

Adjusted for gender, age, family allergy history, annual household income, outdoor PM_10_ and home location. Forward conditional method is applied. ^a^ Floor moisture in child’s room: yes vs. no (ref.). ^b^ Condensation on windowpane in winter: yes vs. no (ref.). ^c^ Perceived moldy odor in room: yes vs. no (ref.). ^d^ Perceived dry air in room: yes vs. no (ref.). ^e^ Floor covering: modern floor covering (linoleum, poly vinyl chloride (PVC), carpets, wood, laminated wood) vs. traditional covering (stone or cement) (ref.). ^f^ Home renovation during child’s early life: yes vs. no (ref.). ^g^ Parents smoking during child’s early life: yes vs. no (ref.). ^h^ Type of childcare: at daycare vs. at home (ref.). ^i^ Size of the class at daycare center: >30 children vs. <30 children (ref.). ^j^ Sun-curing bedding: not often vs. often (ref.). ^k^ Mode of delivery: cesarean delivery vs. natural delivery (ref.). ^l^ Born on due week: no vs. yes (ref.). ^m^ Data is restricted to preschool children.

**Table 6:** Population attributable fractions of home environmental, lifestyle, and biological factors for respiratory infections among children in Tianjin, China.

	Croup, %	Pneumonia, %	Ear Infection, %	Common Cold >2 Times ^a^, %
Floor moisture			0.9	1.4
Condensation	12.2			8.4
Perceived moldy odor	7.6			
Perceived dry air		13.9		15.0
Modern floor covering	14.7	18.3	34.5	
Renovation, early	4.2			
Environmental tobacco smoke (ETS) exposure, early		4.5		
Childcare at daycare center				14.8
High occupancy daycare center			11.8	9.0
Infrequent sun-curing bedding		18.7	28.4	
Cesarean delivery		13.8		
Not born on due week		7.4		

^a^ Data is restricted to preschool children.

## Supplementary Materials

The following are available online at https://www.mdpi.com/1660-4601/17/11/4069/s1, Questionnaire: China-Child-Home-Health, Figure S1: Locations of Tianjin metropolis and Cangzhou city, China, Table S1: Outdoor PM_10_ concentrations in Tianjin area (i.e., Tianjin metropolis and Cangzhou city) in 2013–2014, μg/m^3^ Table S2: Associations between building characteristics and respiratory infections among children, Table S3: Associations between environmental tobacco smoke exposure, pet-keeping and respiratory infections among children, Table S4: Associations between daycare and respiratory infections among children, Table S5: Correlation coefficients calculated by Kendall’s tau- beta test.

## Abbreviations

CCHH	China, Children, Homes and Health
TB	Tuberculosis
ETS	Environmental tobacco smoke
PAF	Population attributable fractions
AC	Air conditioner
AOR	Adjusted odds ratio
CI	Confidence interval
PM10	Particles smaller than 10 microns
PVC	Poly vinyl chloride
